# Effect of treating *Schistosoma haematobium *infection on *Plasmodium falciparum-specific *antibody responses

**DOI:** 10.1186/1471-2334-8-158

**Published:** 2008-11-17

**Authors:** L Reilly, C Magkrioti, T Mduluza, DR Cavanagh, F Mutapi

**Affiliations:** 1 Institute for Immunology and Infection Research, School of Biological Sciences, Ashworth Laboratories, King's Buildings, University of Edinburgh, West Mains Road, Edinburgh EH9 3JT, UK; 2 Department of Biochemistry, University of Zimbabwe, PO Box 167, Mount Pleasant, Harare, Zimbabwe; 3 Institute of Biology, NCSR Demokritos, Neapoleos and Patriarhou Gregoriou 15310 Agia Paraskevi, Athens, Greece

## Abstract

**Background:**

The overlapping geographical and socio-economic distribution of malaria and helminth infection has led to several studies investigating the immunological and pathological interactions of these parasites. This study focuses on the effect of treating schistosome infections on natural human immune responses directed against plasmodia merozoite surface proteins MSP-1 (DPKMWR, MSP1_19_), and MSP-2 (CH150 and Dd2) which are potential vaccine candidates as well as crude malaria (schizont) and schistosome (whole worm homogenate) proteins.

**Methods:**

IgG1 and IgG3 antibody responses directed against *Schistosoma haematobium *crude adult worm antigen (WWH) and *Plasmodium falciparum *antigens (merozoite surface proteins 1/2 and schizont extract), were measured by enzyme linked immunosorbent assay (ELISA) in 117 Zimbabweans (6–18 years old) exposed to *S. haematobium *and *P. falciparum *infection. These responses were measured before and after anti-helminth treatment with praziquantel to determine the effects of treatment on anti-plasmodial/schistosome responses.

**Results:**

There were no significant associations between antibody responses (IgG1/IgG3) directed against *P. falciparum *and schistosomes before treatment. Six weeks after schistosome treatment there were significant changes in levels of IgG1 directed against schistosome crude antigens, plasmodia crude antigens, MSP-1_19_, MSP-2 (Dd2), and in IgG3 directed against MSP-1_19_. However, only changes in anti-schistosome IgG1 were attributable to the anti-helminth treatment.

**Conclusion:**

There was no association between anti-*P. falciparum *and *S. haematobium antibody *responses in this population and *a*nti-helminth treatment affected only anti-schistosome responses and not responses against plasmodia crude antigens or MSP-1 and -2 vaccine candidates.

## Background

Every 40 seconds a child dies of malaria, a total of more than 2000 deaths per day, [1]. Malaria is the most important human parasitic disease in terms of deaths, clinical cases and long term consequences for affected communities. In contrast, schistosomiasis is responsible for relatively few deaths, but is associated with considerable morbidity. About 200 million people are currently thought to be infected, with a further 600 million at risk of infection [2]. The geographical and socio-economic distribution of *Plasmodium falciparum *infection overlaps with that of many helminth infections including *Schistosoma haematobium *in sub-Saharan Africa. This gives potential for interaction in the overall susceptibility, pathology or clinical manifestations for these infections. Indeed there is now a growing body of evidence showing that in both natural and experimental infections, schistosome and plasmodia infections profoundly affect each other immunologically and in the degree of pathology they cause in the host. Several studies have reported both cellular and humoral immunological interactions between plasmodia and schistosome infection [3-6] which may partly be explained by the existence of cross reactive epitopes between schistosome and plasmodia antigens [7,8].

We are interested in the potential effect of schistosome control programmes on malaria vaccine efficacy. Schistosome infection is controlled by treatment of infected people with the anti-helminth drug praziquantel and we have previously shown that treating *S. haematobium *infection alters schistosome specific humoral and cellular responses, accelerating the development of these responses [9,10]. We have subsequently shown that this modulation of immune responses is related to a change in both the quantity and type of antigens recognized by the host's immune system [11]. Since praziquantel treatment is used in people exposed to both malaria and schistosomiasis, it is important to determine if this chemotherapy alters any of the responses to malaria vaccine candidate antigens and therefore inadvertently affects their use for vaccination. Therefore, the aim of this study is to determine whether praziquantel treatment for schistosomiasis modulates natural antibody responses to malaria vaccine candidate merozoite surface proteins [12,13]. We focuses on IgG1 and IgG3 immune responses associated with protection against these antigens [12,14].

## Methods

### Parasite antigens

Lyophilized soluble *S. haematobium *adult worm antigen (SWAP) was obtained from the Theodor Bilharz Institute (Egypt) and reconstituted as previously described elsewhere [11]. The parasite strain is one used for previous immuno-epidemiology studies [15]. Crude *P. falciparum *schizont antigen preparations were a gift of Dr. P. Druilhe, Institute Pasteur, Paris. The merozoite surface protein antigens used were prepared as recombinant proteins in *Escherichia coli*. Two antigens derived from merozoite surface protein 1 (MSP-1), DPKMWR and MSP-1_19 _antigens also known as p190, gp195, [16] were used. MSP-1 can be divided into 17 distinct blocks, based on its conserved, semi-conserved and variable regions. Block 2 is highly polymorphic, with over 50 sequences described. However these sequences fall into one of 3 categories or serotypes: K1; Mad20; and RO33 [17] and DPKMWR is a recombinant protein composed of Block 2 sequences from all 3 block 2 serotypes. MSP-1_19 _is the conserved C terminal of MSP-1, and is the only part to remain on the surface of the merozoite, with the rest of the protein being shed upon erythrocyte invasion. Two full-length recombinant MSP-2 antigens were also used, namely CH150/9 (5/6) and Dd2 (13/14). MSP-2 has 2 serotypes: CH150/9 5/6 is taken from serotype A (3D7-like), and Dd2 belongs to serotype B (FC27-like).

### Study population and sample collection

The study was conducted in the Mashonaland East Province of Zimbabwe (31°30'E; 17°45'S) where *S. haematobium *and *P. falciparum *are endemic. The study area is described in detail elsewhere [18]. Briefly the area is rural with the majority of the people living in thatched houses made up of clay. The main activity in these villages is subsistence farming and human water contact is frequent with at least 4 contacts/person/week due to insufficient safe drinking water and sanitation facilities (see [18] for studies in neighboring villages). Drinking water is collected from open wells while bathing and washing is conducted in two main rivers in the villages. Most families maintain a garden located near the river where water is collected for watering the crops. The villages were selected because health surveys regularly conducted in the region by the Provincial Medical Director showed little or no infection with other helminths and a low *S. mansoni *prevalence (<5%). Permission to conduct the work in this province was obtained from the Provincial Medical Director and ethical approval received from the Medical Research Council of Zimbabwe . Informed consent (following explanation of the study aims and procedures) was obtained before the collection of parasitology and blood samples. The selected villages had not been included in the National Schistosome Control Program and there were no active malaria vector control programmes in the area. Therefore participants had not received treatment for schistosomiasis or other helminth infections meaning that we could study natural immune responses in the absence of drug-altered schistosome responses [9,19].

*Plasmodium falciparum *is the predominant species of malaria in Zimbabwe [20] where malaria transmission is largely unstable in nature. Approximately 5.5 million people out of a total population of 12.7 million live in malarious areas [21]. Out of the 56 districts in Zimbabwe, malaria transmission occurs in 42. In 2002, a revised stratification based on a national parasite prevalence survey, HMIS data, entomological data and expert opinion was prepared which classified our study area under the sporadic transmission regions with low transmission [21-23] meaning that this is a mesoendemic area for malaria [24]. The peak *P. falciparum *transmission occurs from February to May [24] and our sampling times were in March and then 6 weeks later in April. The schools surveyed (a secondary school and its feeder primary school (i.e. where the majority of the primary school children come from) Goromonzi and Shangure Schools in Village, and Chindenga and Nyambanje Schools in Mutoko Village) were all in close proximity to rivers. Stool and urine specimens were assayed for *S. haematobium, S. mansoni *and geo-helminths using standard procedures [25,26] and the intensities of *S. haematobium *calculated from at least 2 urine samples (maximum 3) collected on consecutive days, and those of *S. mansoni *calculated from mean intensities of at least 4 Kato-Katz slides (maximum 6), 2 from each of the 2–3 stool samples collected. Anti-helminth treatment was administered to all compliant people after the first survey as well as 6 weeks later at the end of the study. In order to be included in the cohort, participants had to meet all the following criteria at the two sampling points (pretreatment and 6 weeks post-treatment): 1) have provided at least 2 urine and 2 stool samples on 3 consecutive days which are used for *S. haematobium *and intestinal helminths detection including *S. mansoni*; 2) be negative for intestinal helminths including *S. mansoni *(no one was excluded on this criteria as everyone was negative for these infections), 3) be negative for schistosome infection at the 6 week survey if they had received ant-helminth treatment, 4) have given at least 10 mls of blood for serological assays and preparation of a thick smear slide for microscopic detection of *Plasmodium *parasites. 117 people (53 male, 64 female) aged between 6–18 years old met these criteria and were included in the study. Of these, 89 people received praziquantel treatment while 28 people were not treated either because they did not take western medicine for religious reasons (23) or were not present on treatment days. The 28 people agreed to take part in the study and effectively became untreated controls. Thus the population was stratified by its schistosome and malaria staus as well as schistosome treatment history as shown in table 1. People presenting with malaria were treated according to the treatment regime prescribed by the Ministry of Health in Zimbabwe.

### Immunological assays

The antibody subclasses associated with protection against the MSP antigens are IgG1 and IgG3 [12,14]. Studies in *S. haematobium *have also associated IgG1 and IgG3 subclasses with protection against schistosomiasis [9,27]. Therefore this study focused on these two responses directed against crude schistosome worm antigen (WWH), crude malaria schizont and MSP-1/2 antigens. Indirect enzyme linked immunosorbent assays (ELISA) already published for *S. haematobium *[15] and for the *P. falciparum *antigens [28-30] were used for the assays. Briefly, ELISAs were conducted in duplicate for each sample using ELISA plates (Nunc-Immulon, Denmark) that were coated with 50 ul/well of 50 ng/ml antigen for recombinant antigens and 1 ug/ml for both crude antigens in 60 mM carbonate-bicarbonate buffer (pH 9.6) and incubated overnight at 4°C. Plates were blocked with 200 μl/well of skimmed milk (5% milk in phosphate buffered saline (PBS)/0.03% Tween 20) for 1 hr and washed three times in PBS/Tween 20, which was used for all washes. 100 μl of serum was added to each well at 1:100 dilution for all assays except for MSP1_19 _which was 1:200; plates were incubated overnight at 4°C and then washed three times. 100 μl of subclass-specific monoclonal antibody was added at 1:1000 dilution for the detection of IgG1 and IgG3 (The Binding Site, UK). Plates were incubated overnight at 4°C, washed six times and 100 μl of ABTS substrate solution (KPL, Canada) was added, before the absorbance was read at 405 nm. Three negative controls from European volunteers who had never traveled to malaria or schistosome endemic areas were used on each plate and people were considered reactive if they had an absorbance value greater than the mean plus 2 standard deviations of the negative controls.

### Statistical analysis

Statistical analyses were conducted using the statistical package SPSS and were used to test three distinct hypotheses, 1) *P. falciparum *status is not associated with schistosome-specific responses or schistosome infection level, 2) schistosome infection intensity is not related to plasmodia-specific responses or *P. falciparum *status, 3) treatment of schistosome-infected people with praziquantel does not affect plasmodia-specific responses.

For all statistical analyses, participants were divided into three age groups, 6–11 years old, 12–13 years, 14–18 years old reflecting the epidemiological groupings where schistosome infection levels are rising, peaking and declining as well as give large enough sample sizes for the statistical analyses. The initial statistical procedure was conducted to reduce the number of variables used in hypothesis testing. Partial correlation analysis was used to explore correlations between antibody levels directed against schistosome and plasmodia antigens. This was followed by factor analysis using principal components (PCA) [31]. PCA is a standard technique for reducing multivariate data down to its main independent features [31]. Components are extracted according to the amount of variation in the data they explain so the first component explains the most variation and each subsequent component is included if it explains a significant amount of variation present with the data [32]. This procedure was used in this instance for two reasons (1) to avoid type I and type II errors in multiple tests and using correlated independent variables [33,34] and (2) to determine if anti-plasmodial and anti-schistosome responses clustered separately. Following the factor analysis principal components were analyzed further with logistic regression to determine the risk factors associated with being infected with malaria to specifically address hypothesis 1. The PCA were also used in an analysis of variance (ANOVA) to address hypothesis 2. For this analysis the following variables were categorical; sex (male, female); age (3 categories already described); malaria status (positive, negative) and the PCA factors 1 and 2 as continuous variables. Schistosome infection intensity (log (x+1) transformed) as the dependent variable.

To test the effects of treatment on immune responses it was necessary to first establish if there had been a significant change in antibody responses between the two time points. A non parametric 2-tailed Wilcoxon test for related samples was used for this analysis since antibody levels were not normally distributed. This was followed by an analysis determining if the observed differences were associated with praziquantel treatment, comparing the change in treated vs. untreated people after allowing for the effects of host sex, age, pre-treatment schistosome infection intensity and pre-treatment malaria infection status using repeated measures ANOVA. All statistical analyses were performed using the statistical software SPSS, and type III sums of squares were used to calculate the F-value where appropriate and the p value was set at 0.05 (see [33]). Unless otherwise stated, all statistical procedures used the following variables as categorical; sex (male, female); age (3 categories already described); malaria status (positive, negative); and schistosome treatment (treated, untreated). The following variables were continuous; mean schistosome infection intensity (log_10 _(x+1) transformed), antibody level (absorbance at 405 nm) and the principle components.

## Results

### Schistosome epidemiology

**Figure 1:**
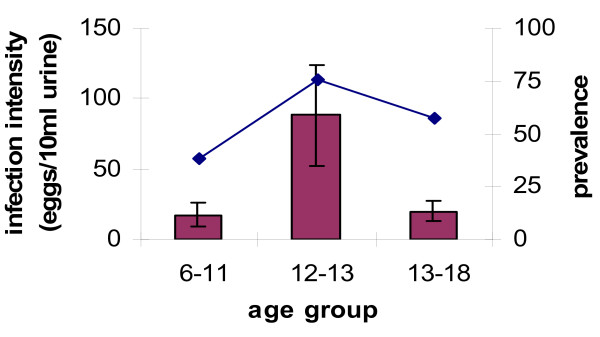
***S. haematobium*****infection intensity (mean of the age group) and prevalence for the different age groups.** Infection rises with age peaking in 12–13 year olds. Bars represent standard error of the mean.

Parasitological examination gave an overall schistosome infection prevalence of 59% and a mean infection intensity of 39 eggs/10 ml of urine (SE = 11 with a range between 0 and 1000 eggs) before praziquantel treatment. Infection prevalence and intensity followed a convex age-infection profile with infection levels rising to peak in children 12–13 years old and infection intensity declining faster than infection prevalence thereafter (Figure 1). Eighty-nine people received praziquantel treatment and 28 were not treated. The prevalence of schistosome infection in this group did not differ significantly (57% and 60% respectively), but infection intensity was higher in the treated group (45 vs. 19 eggs/10 ml urine). This difference was accounted for by allowing for the effects of infection intensity before testing for the effects of treatment on the change in antibody level in the statistical analyses. All participants except 1 showed a detectable anti-schistosome antibody response.

### Plasmodium epidemiology

**Figure 2:**
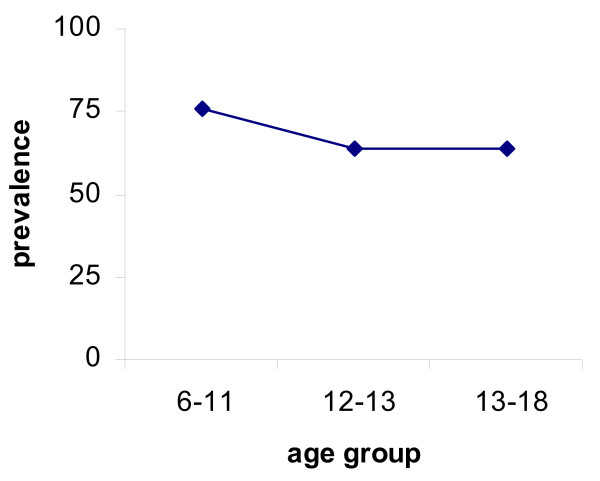
***P. falciparum *****infection prevalence for the different age groups. **Infection peaks in the youngest age groups 6–11 year olds.

**Figure 3:**
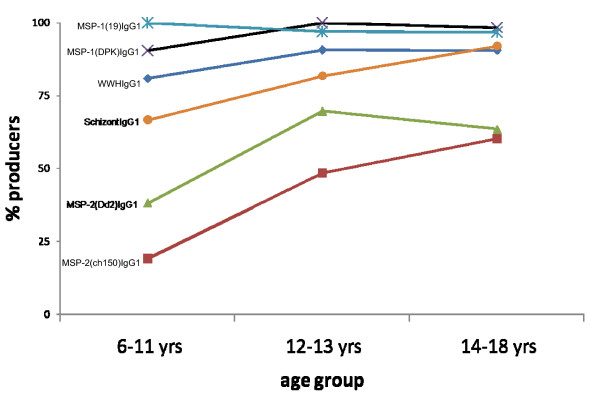
Percentage of people producing detectable IgG1 antibodies against the antigens schistosome WWH (diamond), MSP-2 (CH150)(squares), MSP-2(Dd2)(triangles), MSP1(DPK) (crosses), MSP-1(19) (stars) and *P. falciparum *schizont (circles).

**Figure 4:**
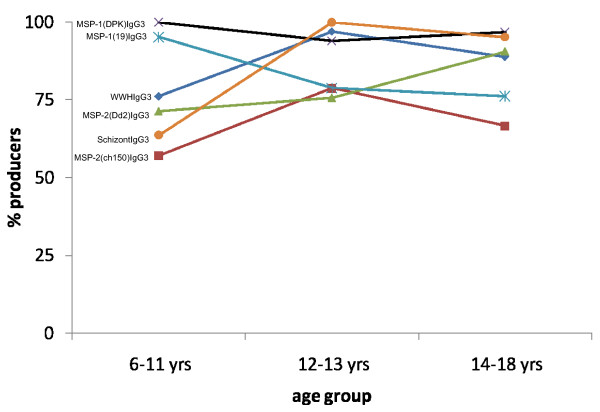
Percentage of people producing detectable IgG3 antibodies against the antigens schistosome WWH (diamond), MSP-2 (CH150)(squares), MSP-2(Dd2)(triangles), MSP1(DPK) (crosses), MSP-1(19) (stars) and *P. falciparum *schizont (circles).

Malaria infection as determined by microscopic examination of thick smear slides was *P. falciparum *and had a prevalence of 66%. Parasite densities were not determined. *P. falciparum *prevalence peaked earlier (in 6–11 year olds) than schistosome prevalence (Figure 2) and did not differ significantly between people who received anti-helminth treatment and those who remained untreated (65% and 68% respectively). *P. falciparum *positive participants were parasitemic but asymptomatic and none of the participants suffered malaria attacks at the time of sample collection or were treated for malaria in between sampling times. There was no significant change in *P. falciparum *prevalence between the two sampling time points (66% vs. 62%) but a few people moved between infection status; 36 people remained negative, 68 positive while 9 people changed status from positive to negative and 4 from negative to positive. All participants except 3 had a detectable IgG3 response *to P. falciparum *schizont antigen a marker for relative exposure levels to *P. falciparum *infection [35] and these 3 did have a detectable IgG1 response against this antigen. For all antigens (plasmodia and schistosome specific), the most prevalent response was IgG3 and this showed different patterns with age to the IgG1 (Figures 3 and 4).

### Immune responses before treatment

**Figure 5:**
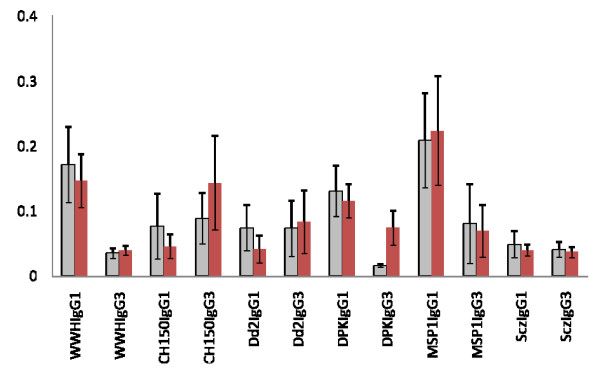
**Mean IgG1 and IgG3 antibody responses directed against *P. falciparum *and schistosome antigens in the group not receiving anti-helminth treatment at the 2 time points. **Bars represent standard error of the mean. Light histograms represent antibody levels at the start of the study, dark histograms antibody values 6 weeks later.

**Figure 6:**
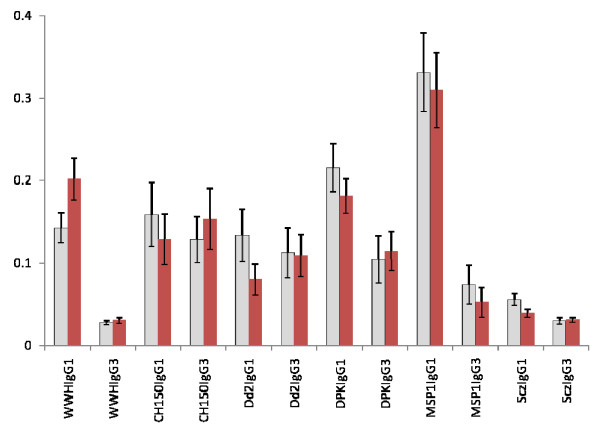
**Mean IgG1 and IgG3 antibody responses directed against *P. falciparum *and schistosome antigens in the group receiving anti-helminth treatment at the 2 time points (i.e. pre- and post-treatment). **Bars represent standard error of the mean. Light histograms represent antibody levels at the start of the study, dark histograms antibody values 6 weeks later (after praziquantel treatment).

The participants mounted immune responses to both plasmodia and schistosome antigens as shown in Figures 3 and 4. There were no significant differences in responses between the two groups (those to receive treatment and those to remain untreated) at the beginning of the study before anti-helminth treatment (Figures 5 and 6). When measuring the levels of antibody responses, those directed against the schistosome whole worm crude antigen were predominantly IgG1 while the responses to malaria schizont antigen were mixed both before treatment and 6 weeks later. Responses to the MSP-1 antigens were predominantly IgG1 and while those directed against the MSP-2 antigens were mixed.

Initial partial correlation analyses controlling for sex, age, malaria status and pre-treatment schistosome infection intensity showed no significant correlations between anti-plasmodial and anti-schistosome responses. This was confirmed by factor analysis which reduced the 12 antibody responses (IgG1 and IgG3 directed against the 6 antigens; WWH, schizont, MSP-1 antigens (DPKMWR, MSP-1_19_), MSP-2 antigens (CH150 and Dd2) into two components partitioned by parasite species with all the anti-plasmodial responses falling in one component (PCA1) and the two anti-schistosome responses falling in a separate component (PCA2) as shown in Table 2. The next step was to determine if these two variables representing anti-schistosome and anti-plasmodial responses affected the risk of a person being positive for malaria infection. This was conducted using a logistic regression which included host age, sex and schistosome infection intensity. This analysis showed that host sex, age and of level of anti-plasmodial/schistosome antibodies did not affect the risk of *P. falciparum *infection. However, schistosome infection intensity was a significant risk factor with the likelihood of being parasitemic for *P. falciparum *increasing with increasing schistosome infection intensity (B = 0.589, SE = 0.270, Wald = 4.77, df = 1, p = 0.029). Conversely schistosome infection intensity was significantly higher in people who were parasitemic for *P. falciparum *(F = 5, 67, df = 1, 117, p = 0.019) and was positively associated with PCA2 (F = 21.5, df = 1,117, p < 0.001). The later means that schistosome infection intensity increased with increasing levels of anti-WWH IgG1 and IgG3. There was no significant association between anti-plasmodial responses (PCA1) and schistosome infection intensity (F= 0.002, df = 1, 117, p = 0.969).

**Table 2:** Results of the Factor Analysis using Principle Components giving the loadings of each antibody response in each component.

	***Principle Component 1 ****(anti-plasmodial responses)*	***Principle Component 2 ****(anti-schistosome responses)*
Initial Eigen values % variance	46.465	13.241
**Variables**	**Loadings**	**Loadings**
WWH-IgG1	0.08	**0.796**
WWH-IgG3	-0.011	**0.879**
MSP-2 (CH150)-IgG1	**0.864**	-0.064
MSP-2 (CH150)-IgG3	**0.715**	0.101
MSP-2 (Dd2)-IgG1	**0.740**	-0.001
MSP-2 (Dd2)-IgG3	**0.857**	-0.30
MSP-1 (DPK)-IgG1	**0.615**	-0.304
MSP-1 (DPK)-IgG3	**0.662**	0.216
MSP-1 (MSP1_19_)-IgG1	**0.682**	-0.2
MSP-1 (MSP1_19_)-IgG3	**0.876**	-0.043
Schizont-IgG1	**0.697**	0.028
Schisont-IgG3	**0.704**	0.211

Principle Component 1 (PCA1) is most influenced by the anti-plasmodial responses while PCA2 is most influenced by anti-schistosome responses. The loadings indicate the correlation between the original variable and the new principle competent (PC), i.e. they indicate the extent to which the original variables are influential in forming the PC [32]. Variables with loadings > 0.5 are highlighted in bold.

### Effect of treatment on anti-schistosome and anti-plasmodial IgG1 and IgG3 responses

**Figure 7:**
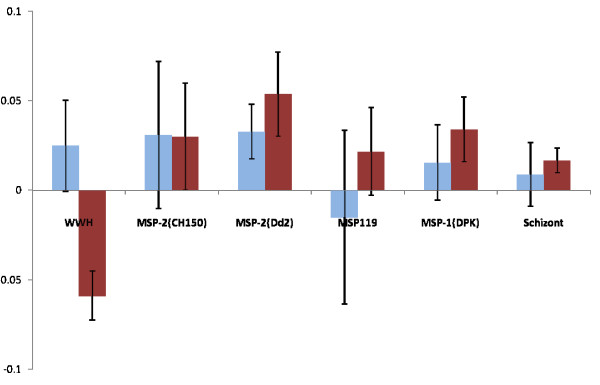
**Changes in IgG1 responses directed against schistosome and *P. falciparum *antigens.** Since these are obtained by subtracting post-treatment levels from pre-treatment levels, increases in antibody levels after the 6 weeks appear on the negative scale, while decreases appear on the positive scale. * represents a significant difference between praziquantel-treated and untreated people at p < 0.05. Bars represent standard error of the mean. Light histograms represent untreated participants, dark histograms represent untreated participants.

**Figure 8:**
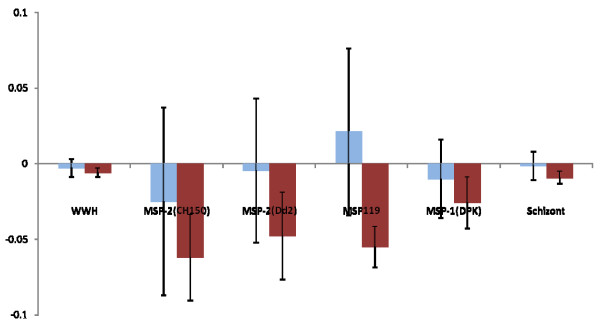
**Changes in IgG3 responses directed against schistosome and *P. falciparum *antigens.** Since these are obtained by subtracting post-treatment levels from pre-treatment levels, increases in antibody levels after the 6 weeks appear on the negative scale, while decreases appear on the positive scale. Bars represent standard error of the mean. Light histograms represent untreated participants, dark histograms represent untreated participants.

Six weeks after treatment of schistosome infections with the drug praziquantel, anti-schistosome and anti-plasmodial responses were assessed again to determine the factors associated with the change in the levels of these responses. There were significant changes in levels of IgG1 directed against WWH, MSP-2 (Dd2), MSP-1_19 _and schizont antigen (Table 3) and in IgG3 directed against MSP-1_19_. Where significant changes occurred, for them to be associated with praziquantel treatment, they had to differ significantly between praziquantel-treated people and the untreated controls after allowing for all other confounding variables (sex, age, *P. falciparum *status and pre-treatment schistosome infection intensity). Only changes in anti-schistosome IgG1 differed significantly between treated and untreated people (Table 3), declining in untreated people, while increasing in treated people (Figures 7 and 8).

**Table 3:** Results of the statistical tests determining if there was a significant change in antibody level between the two time points (pre and post-treatment) and subsequently if any significant changes were related to praziquantel treatment

	***T-test***		***Multi-variate analysis***	
**Response**	**Z-value**	**P-value (2 tailed)**	**F-value**	**P-value**

WWH-IgG1	-3.424	**0.001**	4.083	**0.046**
WWH-IgG3	-1.206	0.228	0.054	0.816
MSP2 (CH150)-IgG1	-0.927	0.354	0.012	0.914
MSP2 (CH150)-IgG3	-1.658	0.098	0.068	0.974
MSP2 (Dd2)-IgG1	-3.386	**0.001**	0.135	0.714
MSP2 (Dd2)-IgG3	-0.895	0.371	0.849	0.359
MSP1 (DPK)-IgG1	-0.738	0.460	0.094	0.760
MSP1 (DPK)-IgG3	-1.885	0.059	0.676	0.413
MSP1 (MSP1_19_)-IgG1	-3.292	**0.001**	0.524	0.471
MSP1 (MSP1_19_)-IgG3	-3.927	**0.000**	0.069	0.793
Schizont-IgG1	-2.342	**0.019**	0.018	0.894
Schizont-IgG3	-0.970	0.332	0.227	0.635

A 2-tailed Wilcoxon nonparametric test for related samples was used to determining if there was a difference between the two time points (antibody data were not normally distributed even after transformations). A multi-variate analysis of variance was used to determined if any significant differences between the two time points were related to treatment. Residuals from this parametric test were normally distributed. The degrees of freedom for this were 1 and 117. For both tests P-values < 0.05 are highlighted in bold.

## Discussion

The overlapping geographical and socio-economic distribution of *P. falciparum *and helminth infections has led to studies investigating the immunological and pathological interactions of these parasites some of which have reported the presence of cross reactive antigens between schistosome and malaria parasites (see [7] for review). There are also several studies which have reported that treatment of schistosome-infected/exposed people with the anti-helminth drug praziquantel alters their immune responses (see [36] for review). Given that there are now several control programmes distributing praziquantel in mass treatment programmes in Africa such as those conducted by the Schistosome Control Initiative , it is vital to determine if such treatment has an impact on natural immune responses directed against malaria vaccine candidates.

In this study IgG1 and IgG3 responses directed against *S. haematobium *and *P. falciparum *antigens were studied before and after anti-helminth treatment with praziquantel. The presence of antibody responses to both *P. falciparum *and schistosome crude antigens (except one person who showed no reaction to schistosome antigens) indicated that the participants had been exposed to the infections and the IgG subclass responses to both parasites were largely in agreement with previously published results. The predominant response to schistosome crude antigens was IgG1 which is consistent with studies in other Zimbabwean populations [15]. Mixed IgG1 and IgG3 responses to plasmodia MSP-2 such as those observed here are common in semi-immune or non-immune cohorts [37]. Responses to the N-terminal Block 2 region of MSP-1 are predominately of the IgG3 subclass, so the mixed responses to the DPK antigen are unusual; this might be explained by the fact that the DPK antigen also includes the Block 1 region of MSP-1 [38], for which previous antibody isotype analysis is not available. As reported in other studies, the subclass response to MSP-1_19 _was biased towards IgG1, with a minor component from the IgG3 subclass [39,40].

In this study the anti-plasmodial responses were not related to the schistosome responses. We and other groups have previously reported correlations between *P. falciparum *and schistosome immune responses. Our previous work suggested that there was an association between immune responses directed against crude antigens from the parasites [41], while Naus *et al *reported cross reactivity between IgG3 directed against *P. falciparum *MSP-2 and *S. mansoni *adult worm antigens in people exposed to both parasites [8]. They suggested that rather than having cross-reactive epitopes the two antigens might have molecules such as lectins that are cross-reactive. In this current study we found no association between anti-schistosome WWH and anti- MSP-2 responses. The differences in our results and those previously reported may be due to use of specific defined antigens as opposed to crude antigens [41]. In cases where defined single antigens have been used [8], the difference in our results may be due to genetic differences in the *Plasmodium *parasite strains which may differ in the repeat sequences of the two MSP-2 antigens used [42,43]. We also showed no association between responses against MSP-1 and schistosome adult worms. This, together with our previous study showing that there was no association between schistosome-specific antibodies with responses directed against MSP-3b [41], suggests that there may not be any cross-reactive epitopes between these *P. falciparum *MSP proteins and schistosome crude antigens. This study showed that the anti-plasmodial responses did not affect the risk of being positive for infection. More detailed studies relating the level of *P. falciparum *parasitemia to these responses are needed to determine if their effect is quantitative rather than qualitative. The oberservation that schistosome infection intensity was a significant risk factor for being *P. falciparum *positive with the likelihood of having malaria increasing with increasing schistosome infection intensity is consistent with findings from other studies [44]. The reason for this association is yet to be elucidated and may include several factors such as host genetics, host exposure patterns and parasite biology.

Anti-helminth treatment increased the level of the anti-schistosome IgG1 response. Although this increase in IgG1 is marginally significant (p = 0.046), it is consistent with previous reports [9] and is not surprising as praziquantel treatment introduces a large amount of parasite antigens to the host simultaneously [45]. In a previous study we reported that there were significant changes in responses directed against MSP-3b and Glutamate Rich Protein (Glurp R0) following praziquantel treatment, but we could not attribute this change to the anti-helminth treatment due to lack of untreated controls in the study [41]. In this current study we were able to include a group of untreated people so that we could explicitly test the effects of treatment on plasmodia and schistosome-specific responses and although there were significant changes in the anti-plasmodial responses none of these were attributable to the anti-helminth treatment. One possible explanation for the changes in the plasmodia responses over the 6 weeks could be related to the dynamics of the *P. falciparum *infection in the study population. Generally levels of IgG1 declined over the 6 weeks while IgG3 levels rose. A recent study in Kenyan children reported that the anti-plasmodial IgG1 and IgG3 responses to MSP-1 and MSP-2 antigens had a short half life with changes detectable within 6 week [46]. In the Kenyan study, levels of antibodies declined after a malaria attack unless they were boosted by the appearance of plasmodia parasites in the blood. In our study most children who were parasitemic at the beginning of the study were also parasitemic at the 6 week time point so that the antigen challenge was present at the two time points. This would have resulted in the maintenance of responses to the antigens and may be indicating the previously published shift from IgG1 to IgG3 responses directed against merozoite surface proteins [37]. Given the mesondemic transmission patterns of the region, full interpretation of the treatment-independent changes requires quantitative studies enumerating the parasites as well as genotyping studies indicating infection/re-infection episodes in the intervening time. However since there were no significant differences attributable to praziquantel treatment in the participants it can be concluded that anti-helminth treatment of people exposed to both *P. falciparum *and *S. haematobium *parasites did not affect antibody responses against the MSP-1 and -2 malaria vaccine candidates. Further studies in a younger age group (children below 5 years of age, (who may be the main vaccine target group ) over a longer period will be valuable in developing integrated malaria and schistosome control programmes.

## Competing interests

The authors declare that they have no competing interests.

## Authors' contributions

LR and CM conducted the immunological assays and preliminary data analyses supervised by FM and DC. They also prepared the first drafts of the manuscript. DC provided *P. falciparum *antigen expression plasmids and LR and DC prepared the recombinant *P. falciparum *antigens. FM and TM conducted the field work and developed the hypotheses for testing. All authors read and corrected draft copies of the manuscript and approved the final version.

**Table 1:** Sample sizes of the population classified by schistosome and *P. falciparum *infection status

	***Praziquantel Treated***		***Untreated controls***	
	*P. falciparum *parasitemia positive	*P. falciparum *parasitemia negative	*P. falciparum *parasitemia positive	*P. falciparum *parasitemia negative

***S. haematobium *egg positive**	36	17	11	5
***S. haematobium *egg positive**	22	14	8	4

## Pre-publication history

The pre-publication history for this paper can be accessed here:


